# Conceptualising the empowerment of caregivers raising children with developmental disabilities in Ethiopia: a qualitative study

**DOI:** 10.1186/s12913-023-10428-4

**Published:** 2023-12-15

**Authors:** Zsofia Szlamka, Ikram Ahmed, Elisa Genovesi, Mersha Kinfe, Rosa A. Hoekstra, Charlotte Hanlon

**Affiliations:** 1 Department of Psychology, Institute of Psychiatry, Psychology & Neuroscience, King’s College London, London, UK; 2 Care Policy and Evaluation Centre, London School of Economics and Political Science, Houghton Street, London, WC2A 2AE UK; 3 Department of Health Service and Population Research, Institute of Psychiatry, Centre for Global Mental Health, Psychology & Neuroscience, King’s College London, London, UK; 4 Department of Psychiatry, School of Medicine, WHO Collaborating Centre for Mental Health Research and Capacity-Building, College of Health Sciences, Addis Ababa University, Addis Ababa, Ethiopia; 5 Centre for Innovative Drug Development and Therapeutic Trials for Africa, Addis Ababa University, Addis Ababa, Ethiopia

**Keywords:** Developmental disabilities, Ethiopia, Advocacy, Empowerment, Low- and middle-income countries, Caregiver interventions

## Abstract

**Background:**

Caregivers of children with developmental disabilities (DDs) in Ethiopia experience stigma and exclusion. Due to limited existing services and substantial barriers to accessing care, they often lack support. Caregiver empowerment could help address injustices that hinder their capacity to support their child as they would like. The aim of this study was to explore the meaning and potential role of empowerment for caregivers raising a child with a DD and how empowerment was situated in relation to other priorities in service development.

**Methods:**

This was a qualitative phenomenological study. Semi-structured interviews were conducted in Amharic and English with caregivers of children with a DD (*n* = 15), clinicians (*n* = 11), community-based health extension workers (*n* = 5), representatives of non-governmental organisations working with families with DDs (*n* = 17), and representatives of local authorities in health, education, and social care (*n* = 15). Data were analysed thematically.

**Results:**

Three main themes were developed: “Barriers to exercising caregivers’ agency”; “Whose decision is it to initiate empowerment?”; and “Supporting caregivers through support groups”. Caregiver capacity to do what they thought was best for their child was undermined by poverty, a sense of hopelessness, experience of domestic abuse and multiple burdens experienced by those who were single mothers. Caregivers were nonetheless active in seeking to bring about change for their children. Caregivers and professionals considered support groups to be instrumental in facilitating empowerment. Participants reflected that caregiver-focused interventions could contribute to increasing caregivers’ capacity to exercise their agency. A tension existed between a focus on individualistic notions of empowerment from some professionals compared to a focus on recognising expertise by experience identified as vital by caregivers. Power dynamics in the context of external funding of empowerment programmes could paradoxically disempower.

**Conclusion:**

Caregivers of children with DDs in Ethiopia are disempowered through poverty, stigma, and poor access to information and resources. Shifting power to caregivers and increasing their access to opportunities should be done on their own terms and in response to their prioritised needs.

**Supplementary Information:**

The online version contains supplementary material available at 10.1186/s12913-023-10428-4.

## Background

Caregivers raising children with developmental disabilities (DDs) face various barriers when supporting their child’s optimal development [1, 2]. There is robust evidence from across the globe suggesting that caregivers frequently feel distressed [3, 4], and report a lack of control over their child’s support [5–8]. Evidence indicates that this stress is often a result of lacking treatment options and support; and can be increased in a community where awareness about DDs is low [9]. Caregivers may also go through a process of grieving, especially just after receiving the diagnosis [3, 4]. They often worry about the future of their child, particularly in relation to their education, personal development, and future employability [10, 11]. Another key challenge that caregivers face across contexts is stigma [12–14]. Caregivers are often be exposed to structural stigma due to the lack of organisational and policy support for children with DDs [15], leading to feelings of disempowerment and marginalisation [16].

In Ethiopia, caregivers of children with DDs regularly face stigma and a lack of understanding from their family and community [17, 18]. Ethiopia has a shortage of support services and professionals for children with DDs [19, 20]. The services that do exist are centralised in the capital city of Addis Ababa and are unavailable to most caregivers [20, 21]. Initiatives are underway in Ethiopia to improve services and the quality of life of families raising children with DDs [22]. The Ethiopian government has endorsed the scale-up of the World Health Organization (WHO) Mental Health Gap Action Programme [23]. A key component of this package is the WHO Caregiver Skills Training (CST) intervention [24]. The CST is a low-intensity programme for caregivers of children between two and nine years of age with DDs or with developmental delays which may not (yet) have received a formal diagnosis [25]. The CST consists of nine sessions delivered to groups of caregivers and three home visits, and is manualised by facilitator guides and participant booklets [25]. The CST has been adapted and assessed for feasibility and acceptability within Ethiopia [26]. Ethiopia’s most recent National Mental Health Strategy for 2020–2025 explicitly includes developmental disabilities in its focus area and outlines the empowerment of individuals, families, and population as a key objective, to promote their mental health and protect against risk factors [27].The concept of empowerment entails addressing unequal access to opportunities through a shift in existing power structures [28–30]. Empowerment theories seek to understand what causes powerlessness, and how societal processes can change from top-down control to shared power [31]. There are different views about what outcomes empowerment is expected to achieve. In low- and middle-income country contexts, empowerment is often associated with poverty reduction and holding institutions accountable for public service [32]. Whereas in high-income country settings empowerment is more geared towards strengthening psychological skills and individual-focused empowerment [32]. Most authors agree that empowerment involves increasing the control, agency, and decision-making capacity of an individual, group, or organisation [33–35]. Existing work often differentiates between psychological, economic, and community empowerment [30, 36–38].

In the context of supporting young children with DDs, caregiver empowerment is proposed to address injustices that hinder caregivers’ capacity to support their child [39]. The United Nations’ Convention on the Rights of Persons with Disabilities created a legal basis to empowerment [40] and served as the precursor to interventions combining empowerment with the goal of improving health outcomes for persons with disabilities [41].

Caregiver empowerment could lead to various beneficial outcomes, although existing evidence originates mostly from high-income contexts. For example, empowerment is associated with increased self-efficacy [42] and improved mental health for caregivers, and it may lead to reduced problem behaviours of children with DDs [43]. Caregiver empowerment is also associated with higher service usage, and awareness of existing social support [44]. Empowerment can help caregivers claim their rights, and demand accountability for accessible public services, public policies supporting those with low financial resources, and participation to express their views [45]. There are some studies from low- and middle-income African country settings finding that caregiver empowerment can be a useful concept locally [39, 46, 47], including previous work in Ethiopia [48, 49]. Despite the proposed benefits of empowerment, some authors point to potential problems with the concept of caregiver empowerment [50–52]. Programmes intended to empower can actually be disempowering: for example, a programme seeking to empower women in Ethiopia was critiqued as putting women in an even more disadvantaged position by offering them volunteer work instead of a paid job [53]. In Ethiopia, the potential role of empowerment in developing services for caregivers of children with DDs is not well studied. A clear understanding of empowerment across stakeholder groups involved in supporting families would help to ensure contextual fit and situate empowerment within the development of more people-centred services. While the literature features a range of definitions for empowerment, in this study we used the following working definition to caregiver empowerment:

Empowerment entails a shift in who has control and agency over supporting a family, and specifically a child with a DD. Shifting power towards caregivers means taking a people-centred and rights-based approach in service development. It implies that it is ultimately caregivers whose voices, needs, and priorities that should drive policy, and intervention development.

## Aims

The aim of this study was to explore the meaning and potential role of empowerment for caregivers raising a child with a DD and how empowerment was situated in relation to other priorities in service development. Within the broader class of DDs, we focused on children with cognitive, social or communication delays due to the higher stigma and challenges faced by these children and their families [54]. The research questions were informed by existing literature on power in global mental health [55], empowerment theories [56], and research on caregiver-mediated interventions [57]. The following research questions were studied:

- *What are the perceptions of caregiver empowerment among those raising children with DDs, and those involved in supporting families of children with DDs?*
- *What are the structural barriers to social inclusion and accessing resources, information and support services? How can such barriers be overcome?*

## Methods

This was a phenomenological, qualitative study [58, 59]. When reporting results, we share the perspectives of all informant groups interviewed. We take a people-centred view on empowerment and portray caregivers’ views at the centre, comparing the views of other participant groups against them.

### The study setting: Ethiopia

With a population of around 115 million people in 2020, Ethiopia is the second most populous country on the African continent [60]. Despite a decline in multidimensional poverty in recent years [61], Ethiopia still has one of the highest levels of economic poverty in the world [62]. Ethiopia only spends around 1.4% of its Gross Domestic Product on health care [63], amounting to $26.74 per capita in 2019 [60]. Ethiopians can access public health facilities if they pay into the community-based health insurance system, with fees based on their ability to pay [64]. The poorest families in an administrative unit can theoretically access an exemption certificate [20]. Despite the government’s effort to match healthcare costs with users’ economic capabilities, out of pocket expenditure remains a huge barrier to accessing services [65]. There are no welfare payments for people affected by disability or chronic illness [64]. Ethiopia is a signatory to the United Nations’ Convention on the Rights of Persons with Disabilities [40], however, there is no national legislation on protecting the rights of persons with disabilities [66]. There are three types of school settings in Ethiopia that offer education to children with disabilities: these include special schools (day schools and boarding schools), special units in regular schools and inclusive regular classes [67].

### Participants

#### Recruitment

Participant recruitment took place in three locations: Addis Ababa, the capital city; Bahir Dar, the capital of the Amhara region; and Butajira town in the Gurage zone, about 134 km south of Addis Ababa. We included a variety of locations to explore perspectives across rural and urban settings, to grasp some of the regional differences in Ethiopia, and allow for better transferability of results [68].

Members of the following groups were invited to participate: 1) caregivers of children with a DD; 2) community-based health extension workers; 3) representatives of non-governmental organisations (NGOs) working with families and children with DDs; and 4) representatives of local health, education, and social care authorities.

Our sampling strategy was based on gatekeepers for each stakeholder group [46]. Gatekeepers were identified based on previous research partnerships and collaborations with the research team [1, 20, 69, 70]. Their role was to circulate the call for participation to members of their organisations. We used a snowballing method to reach further participants [71]. Of potential participants contacted, three did not express an interest to participate.

We recruited clinicians and caregivers in three public hospitals in Addis Ababa: Yekatit 12, St Paul and Black Lion Hospitals. Second, caregivers and community-based health extension workers from Butajira were recruited from the participant pool of a previous study on adapting and pre-testing the WHO CST in Ethiopia [26]. Third, study investigators made contact with gatekeepers from NGOs, schools, government ministries, and international organisations working with families with DDs. We also recruited participants directly from these organisations in Addis Ababa and Bahir Dar.

#### Participant demographics

A total of 63 semi-structured interviews were conducted in Amharic (*n* = 19) and English (*n* = 44) between January and March 2020 (see Table 1). Data collection stopped at the point at which no new information occurred (data saturation) [72]. Caregivers could participate without a formal diagnosis for their child. We made this decision based on the inclusion criteria set in existing work, such as in the WHO CST [26]. This practice is informed by the fact that diagnostic services are unavailable to most caregivers in Ethiopia [1, 17]. Overall, twelve caregivers had participated in the WHO CST or a caregiver support group, two were on the waiting list to be offered these services, and one had access to private services. All caregivers who expressed interest in participation were female. The average age of caregivers was 35.4 years. They had on average two children and there was an average of six people living in the household. Professional participants had on average five years of work experience.

**Table 1:** Participant numbers per stakeholder group and location

	**Addis Ababa**	**Butajira**	**Bahir Dar**
Clinicians (psychiatrists, psychologists, nurses)	11		
Community-based health extension workers		5	
Non-governmental organisations/funding or technical support	7		3
Non-governmental organisations/service provider	3		4
School leaders	4		
Special education teachers	3		
Caregivers	10	5	
International organisation representatives	5		
Government representatives	3		

### Study procedures

We developed the interview guides for semi structured interviews with each participant group, informed by previous work on caregiver experiences in Ethiopia [20, 69] and in discussion with the research team. The interview guides for all participants covered the following topics: met and unmet needs of caregivers; caregiver empowerment; experiences with caregiver interventions; experiences with different stakeholder groups offering support for children with DDs.

The phrasing of questions differed across stakeholder groups. For caregivers, we aimed to avoid using technical or potentially stigmatising terms. For example, instead of ‘developmental disabilities’ (the Amharic translation of which has a derogatory connotation) we used the phrase ‘slowly developing children’, as advised by caregiver input into previous studies in Ethiopia. We thought that using the very term ‘empowerment’ might be unfamiliar for caregivers. Hence, we operationalised empowerment in questions such as:

- *Is there someone else you wish you could talk to about your concerns? Would they be able to help? If so, in what way?*
- *Can you tell me about things that you find important to provide your child or family with? Is there something you would like to be able to provide but you cannot at the moment? If so, can you tell me more about this?*
- *Who do you think should know more about children developing slowly? Can you give examples of an organisation or a person?*

In case of professional participants, the interview guides included questions about their beliefs and experiences with empowerment. In their topic guide we used technical terms such as empowerment.

The guides were developed iteratively after each interview in discussion within the research team. For example, we added a question about experiences with inclusion and one about perspectives on inclusive education, because many participants raised that education was a means to support families with children with DDs. We also iteratively added the topic of perspectives on the international presence in Ethiopia’s service development scene. The full interview guides can be found in Additional file 1.

All interviews lasted for up to an hour. English-language interviews were conducted by the first author (ZS), a female PhD Candidate in Psychology at the time of data collection. Prior to data collection, ZS spent two months in Ethiopia to get to know the setting and potential participants. Interviews in Amharic were conducted by Ikram Ahmed (IA), a female Ethiopian clinical psychologist. Both interviewers had had previous experience with qualitative research. Prior to the interview, the researchers explained that the study took place as part of ZS’s PhD programme. Interviews took place at the convenience of participants, in person in their office in case of professionals, or their home in case of caregivers. In case of some of the caregivers, their children were also in the room during the interview. Interviews were audio recorded. If participants did not feel comfortable with recording, the interviewer took notes during the interview – this happened in the case of one interview.

An audio recorder was used to record conversations. Recordings were stored on an encrypted computer. Any personally identifiable data were stored in a password-protected file that only ZS had access to. Once data analysis was completed, audio records were deleted. Following data collection, all interviews were transcribed and those in Amharic were translated to English by a local team of translators. Translators had previously contributed to global mental health-related research.

### Analysis

Data were analysed inductively, using reflexive thematic analysis. Transcripts were imported to and analysed in the qualitative data management software NVivo 12 [73]. We chose thematic analysis to allow for the exploration of the concept of empowerment. First, five randomly selected interviews were coded inductively by ZS and IA. Following the development of an initial codebook, data were analysed iteratively and in discussion within the research team (comprising Ethiopian and international authors). This practice allowed for investigator triangulation (confirming findings across researchers) [74] and minimised the risk of misinterpretation given the lack of sufficient contextual knowledge in case of authors such as ZS, who is not from Ethiopia. Following theme development from initial codes, we also undertook deviant case analysis (identifying outlier perspectives in the study) to enhance the rigour of the study [75]. The final codebook is included in Additional file 2. In our reporting, we followed the COREQ guidelines (the full checklist is attached in Additional file 5) [76].

### Positionality

ZS designed this study from a position that an empowerment approach could be a beneficial way to support caregivers raising children with DDs in Ethiopia, based on existing literature [48, 49]. ZS being a White and European researcher is likely to have had an impact on what participants felt comfortable sharing (please see Additional file 3 for direct quotes on participant perspectives on ZS’s research position). When IA conducted the interviews, some participants referred to her as someone directly involved in the research team adapting and implementing the WHO CST locally [26] and this might have influenced what caregivers felt comfortable sharing. Co-authors previously contributed to research on the lived experience of caregivers of children with developmental disabilities in Ethiopia, highlighting the challenges faced by caregivers [1]. This background may have shaped our interpretations of the data.

### Community and public involvement

This research builds on ongoing research on DDs in Ethiopia, which benefits from continuous input of a project advisory committee. This committee comprises of a range of stakeholders, including caregivers, health and education professionals and representatives from government and non-governmental organisations. The committee advises the research team on research questions, methods, and measures. For this specific study, we consulted clinicians from the hospitals where we recruited caregivers, who provided feedback on the interview guides and data collection methods. We received feedback on the research questions from Ethiopian potential participants of the Autism Advocacy Leadership meeting held in Addis Ababa in January 2020. Preliminary themes and subthemes of the analysis were shared in May 2022 during the Project Advisory Committee meeting of SPARK, a project aiming to improve support for children with DDs and their families in Ethiopia and Kenya. Lastly, a summary of the final themes and subthemes was shared online with participants proficient in the English language (most professional participants, but unfortunately excluding all caregivers). Most participants agreed with the final interpretation of themes. One participant communicated that their organisation was going to focus more heavily on caregiver empowerment, partially inspired by the results of this work.

## Results

We developed three main themes: “Barriers to exercising caregivers’agency”; “Whose responsibility is it to initiate empowerment?”; and “Supporting caregivers through support groups”. Themes and sub-themes are summarised below in Table 2. All themes will be illustrated with quotes from participants, while the full list of relevant quotes are attached in Additional file 4.

**Table 2:** Themes and sub-themes

Theme	Sub-theme
Barriers to exercising caregivers’ agency	Caregivers in poverty
	When caregiving falls on a single mother
Whose responsibility is it to initiative caregiver empowerment?	Caregivers at the centre of empowerment
	The role of professional stakeholders in caregiver empowerment
	Local and international voices in service development
Supporting caregivers through support groups	

### Barriers to exercising caregivers’ agency

Caregivers emphasised that they wished to do everything within their capacity to support their children. However, socio-economic factors such as living in poverty, or raising their child as a single mother meant barriers to exercising their agency – as covered in the two sub-themes below.

#### Caregivers in poverty

Most caregivers mentioned how their choices were constrained by financial struggles, which often made them feel distressed. They all found it difficult to make ends meet and struggled to find a balance between earning an income and finding the time and resources to support their child. For example, some caregivers mentioned that at times their hospital appointment fell on market day, when they could sell goods and earn an income. Financial struggles impacted how caregivers felt they could support their child with a DD – illustrated in the quote below.

*PCP1204, caregiver*

*“I might not take her [child with a DD] to the hospital, she might not see her doctor at the appointed time, or she might interrupt her medication when I have financial problems. I become upset when this happens, and I sometimes say that I shouldn't have given birth to her in the first place.”*

Many caregivers said that an income, stable housing, and access to day care or schooling for their child would allow them to achieve their aspirations and create the living environment they wished for their family. Many professionals added that in their clinical or social care practice they saw how caregivers were desperately trying to find resources for their family. Some mentioned having met families who wished to leave the child who they struggled to support at a hospital or NGO in the hope that the children would then access better resources.

Many women felt upset, stressed, and helpless because of the combined challenges of raising a child with a DD, undermining their sense of agency. Some mentioned that their physical health worsened because of the stress or because they prioritised taking care of their children. A few women expressed that they paid attention to their own physical and mental health predominantly so that they could then further support their child.

*PCP1204, caregiver*

*“I take care of myself because I know that [my child with a DD] needs me. I know that I am more important to her than her father, her aunt, and her brother. Her mother is more important to her than other people. I should take care of myself to take care of her.”*

#### When caregiving falls on a single mother

Single mother participants suggested that the challenges associated with single parenthood further impeded their ability to help their children. Many single mothers could not work while taking care of the child with a DD alone. Others carried their child to work. They shared that the often felt socially excluded and unable to share their challenges with others. Some mentioned that they lacked a support network as they did not have relatives living nearby. Others were afraid of the stigma in the community relating to their status as both single mothers and caregivers raising a child with a DD, and hence preferred not to share their worries with others, illustrated by the quote below.

*PCP0051, caregiver*

*“I deal with them [problems] by myself. If you share your problems with people, they will talk about it with other people. So, I don’t like sharing my secrets with other people. I keep them to myself. I don’t have any relatives. I talk to the priests about my concerns in my daily life because they are better than other people.”*

Many professionals said that DDs were often viewed as a curse or punishment on the family for sins committed by the mother. Many mentioned supporting women whose partner had left because of the child with a DD, because the pregnancy was unplanned, or because fathers believed it was the mother’s fault that the child developed a DD. To illustrate the vicious cycle of challenges that single mothers may experience, a clinician explained how the clinical assessment for DDs may also contribute to mothers being blamed for the child’s DD.

*PCP114, clinician*

*“We ask parents, if the mother, if she was taking any medication, if she was drinking alcohol during pregnancy, it’s just like a formality. Right? It’s a simple question for us to ask. But we won’t notice that mothers will be taking this seriously…So for example, you know, tella [local alcoholic drink] is very common. So, when we ask this kind of questions, so a mother would also think the one glass tella she had, and that can be also like a point of fight or discussion for parents…the husband will be blaming, I was telling you not drink, not to drink that tella, you have been drinking it and so on. So, you know, unconsciously we have been also facilitating this, the blame shifting to mothers, even as professionals...”*

Some of the single mothers were survivors of domestic violence. Some mentioned how they used to feel that they could not ask for help from the community, as speaking negatively about their husband would be discouraged. Others saw their husband’s abusive behaviour as the cause of their child’s DD. Some women decided to separate from their husband to avoid abuse and improve their and their children’s life conditions. Professionals found that there was no infrastructure supporting survivors of violence and that women may not know when and where to ask for help. The quote below illustrates a caregiver’s empowerment journey from experiencing neglect and abuse by her partner to developing an independent life.

*PCP1315, caregiver*

*“He [my ex-husband] ate and drank whatever he liked, and he didn't care about us. He is a health worker, but he didn't care about our health. It was very difficult to live with him. I couldn't visit my family, and I was like a prisoner. We didn't have any social life. The kid should be included in the community and go to school. We didn't even have idir [a burial society helping to save money for funerals and offering social support]. How can I live if I don't go out and mingle with people? It was very difficult to live with him… I don't have any problem now. My life is not luxurious, but I am self-sufficient. Moreover, I had a salary raise recently. Above all, no one abuses me now. I spend as much money as I want for my children and myself, and I save the rest of the money for the future.”*

A few NGO-based professionals highlighted that through supporting women caregivers with education and setting up their own business, they could become less reliant on the husband as a primary breadwinner.

*PCP212, NGO representative*

*“…because you didn't expect a female can be agrarian or more productive in this regard, she is female and at the same time she's disabled. So the community was taking a lesson from her… She is woman, but she is productive. Why not?”*

### Whose responsibility is it to initiate empowerment?

Participants discussed who has the capacity to initiate service development for caregivers and whether that stakeholder is actively engaged in meeting the needs of the beneficiaries. Many participants saw caregivers at the centre of change, as discussed in the first sub-theme. Participants also discussed the role of professional stakeholders in empowerment, discussed in the second sub-theme. Lastly, as covered in the third sub-theme, participants discussing the strong international presence in Ethiopia.

### Caregivers at the centre of change

Most caregivers considered themselves to be the keys to developing the support system for their children. Some women explored advocacy as a tool to access support for their family and started taking a more active role in supporting their children, for example by sharing about DDs in public. Some added that participating in a caregiver intervention facilitated taking this step.

*PCP1003, caregiver*

*“These children [with DDs] didn’t become like that [develop the DD] willingly, and it happened to them by nature. We should teach people that it is not a curse [from God]. Like any human being, these children should be treated equally with typical children. We teach families to raise their awareness of the problem, and we work with aid organizations to solicit their help.”*

Some caregivers discussed their plans to expand existing caregiver support groups. Others added that they were looking for other caregivers who may benefit from help. As the quote below indicates, there were even examples to caregivers who started saving together to be able to financially support their ideas.

*PCP1204, caregiver*

*“Before the new year, we asked the government to enrol our kids in special needs schools. The government doesn’t give quick replies. September passed, and there was some violence after that [political conflict]. Some of the members stopped coming to the meeting. We planned to contribute some money to help each other at the time of death and to support the poor members of our group. If the thirty of us contribute some money, we can lend it to one of the parents when it reaches some amount. That parent could do some business and make a profit. Creating a job opportunity for someone can't be overlooked. We believe that helping the mother means helping the child. God willing, we plan to open a [special needs] school in the future.”*

Many professionals thought that caregivers should be the key decision-makers about what their empowerment should look like. They thought that the goal of caregiver empowerment should be that caregivers feel confident in making decisions about their child’s support, and that they can protect their own wellbeing against stigma. Some mentioned that this empowerment should take place through giving caregivers access to education and resources. A few professionals thought that empowered caregivers should be able to make health-related decisions on their own, as suggested by the quote below.

*PCP515, government representative*

*“So one aim of these kinds of approaches [empowerment] is to make the community knowledgeable and skilled to handle the health issues by themselves with time. So this is empowering the community…empower the community is to handle their own issues, by themselves… The final product, we intend them to…manage their own health.”*

#### The role of professional stakeholders in caregiver empowerment

Many caregivers suggested that while they were key agents in supporting their children, the government was also responsible for providing consistent support to families. Some Community-based health extension workers also suggested that local governments should prioritise the financial support of families with DDs when aid funding was available. Other caregivers thought that the media should take a more active role in raising awareness and breaking the stigma around DDs.

*PCP0935, caregiver*

*“I think that there should be more organisations that help children with similar problems like my daughter. […]I started to openly speak about it only recently. If the mass media speaks about the problem, people will not keep their children locked in the house and they will take them to the hospitals to find solutions for their problem.”*

Some NGO-based participants thought that it was NGOs’ and the government’s role to empower underrepresented groups by providing information on families’ rights and giving skills to manage the child with a DD Community-based health extension workers. Many NGO representatives also shared the view that the needs of persons with disabilities were often addressed only within the remits of health or social care. They suggested that persons with disabilities, caregivers, and professionals should advocate so that disability needs are mainstreamed: considered and implemented in all sectors outside of health and social care.

Many NGO-based participants’ views on empowerment focused on poverty eradication through training those in poverty on business skills and running their own business. Many spoke about caregivers with a child with a DD in the context of microfinance initiatives as a beneficiary, as someone intervened at, someone in the need of external agency. These professionals frequently used technical international development-related vocabulary, including terms such as working on *livelihood programmes* or *income generating activities*. Many shared that they mostly saw people with physical disabilities in these programmes and that people with DDs were underrepresented.

Some professionals in international organisations added that a barrier to caregiver empowerment was when organisations in policymaking power only had a nominal commitment without actionable steps or resources to support families.

*PCP514, international organisation representative*

*“[Our organisation’s commitment to empowerment] it’s like theoretical…I’m just one person working on this [DDs], all of us, we can admit that, we don’t have all that complex competence to [empower], we may have the wish, but we don’t have capacity and also we don’t have the funding. We don’t have the support mechanism.”*

#### Local and international voices in service development

Participants added a further layer to the question as to whose voice counts in service development for DDs when they discussed the strong presence of international organisations and funding bodies in Ethiopia. Some international professionals thought that international service providers were present to support development areas where there would otherwise be no capacity, such as services for children with DDs. However, other international and local professionals thought that international funding created power imbalances in service provision. For example, some local professionals commented that typically, Western stakeholders were in the position of deciding which local project received international funding. An other example mentioned was that if international organisations stopped their funding, entire development areas could fail. Local professionals also suggested that international agencies may be exploiting resources and opportunities in Ethiopia, without appreciating local contributions. Many worried that international organisations may fail to be transparent and communicative about the agenda they lobby for through their funding. Others raised the concern that international professionals may be appointed at leadership positions of international NGOs instead of local staff. They said this was likely the case because international leadership feared corruption and lacked trust at the local level. As the quote below indicates, some professionals even feared that some international actors may look at Ethiopia as a potential for profitmaking. This suggests the potential exploitation of local caregivers.

*PCP114, clinician*

*“There are people coming here knowing that this is a poor country, knowing that little has been done, so people come here to do an investment, I know people in person even, like those who have been certified there so they will come here, they thought they will be the only professionals, so they can earn more money and so on.”*

Power imbalances were also present in the question as to who was appointed to positions of service provision to families with DDs. For example, some Ethiopian professionals shared having met colleagues from abroad who were serving in roles for which they did not have the right level of qualification. Some Ethiopian professionals suggested that blurred professional boundaries and disrespecting caregivers’ knowledge put families who were desperate to find help at additional risk and vulnerability. There were also a few instances where international participants viewed caregiving differently from caregivers themselves, but without necessarily considering existing parenting approaches – as illustrated in the quote below.

*PCP225, NGO representative*

*“…they [the caregivers] don’t learn as much about how to parent a child because when I do the training for the parents, I’m talking about just very basics, discipline, like using timeout…they’re very surprised and they say, wow, we didn’t have this information…So that's the main focus we have with our parenting class, is just teaching the mothers these basic things of how to manage their child at home, it’s really lacking here and then causes a lot of unnecessary stress for the family because the children are just completely out of control.”*

Some local professionals feared that dependency may evolve between international sources and local NGOs and service providers. For example, local clinicians mentioned that materials to support children with DDs, such as information booklets for caregivers, would often come from abroad. They added that these were rarely adapted to the local language and culture. Some NGO representatives raised that when international funding is offered in the form of help, it can be disempowering for local professionals and organisations. Others thought that continuous international funding may mean that the local government never develops a local incentive to fund interventions and services developed.

*PCP215, NGO representative*

*“One of the questions I have often had is who would complain if someone is constantly giving you funding and money for things…who would ever say we don’t want this?... They [the local NGO] know that this [intervention] has been funded by us [international organisation] for so many years. Our expectation would be that eventually they would like it so much, and they would feel so proud of it and they would want ownership of the programme that they would start paying for it. But economically, why would they? If they know someone else is there willing to give them funding for this, what’s the push? To start paying for it on your own, and if that funding leaves, do they feel attached enough? … And even if they believe the government should fund them, does the government believe they should fund these efforts?”*

### Supporting caregivers through support groups

Many informants thought that caregiver support groups could be an effective in increasing caregivers’ ability to exercise their agency. Women who had previously attended support groups described that being around other caregivers who understood what it was like to raise a child with a DD created a shared bonding experience. They said that even if their children may have had different DDs, their experiences as caregivers were similar and this helped them bond. Others added that by meeting caregivers with children with different levels of developmental delays, they may feel less stressed about the development of their own child.

*PCP0935, caregiver*

*“I share my problems with those whom I think could help me solve my problems…. And when I come here [support group], I see many children who have more severe problems [than my daughter]. I tell myself that my daughter is in a better situation than these children, and her only problem is that she has a learning difficulty.”*

Some caregivers added that they wished to proactively support other parents with the resources they had, for example bysharing what they learnt during caregiver programmes with other parents. Others mentioned that caregiver support groups allowed for some caregivers could go to work and take care of household chores. The quote below illustrates a caregiver’s empowerment through her advocacy for DDs and support offered to others.

*PCP0232, caregiver*

*“I am encouraging many people to come and join us [in the caregiver intervention] and enrol their kids in schools. When I meet them in the streets, I tell them that they shouldn't keep their children at home just because they are disruptive. I also tell them that they can keep their children with me.”*

Some clinicians had experiences with caregiver support groups in hospitals. Many thought that support groups could help caregivers feel hopeful about the future opportunities and skills of their child. Many added that explaining to caregivers how and why professionals support the child with a DD is essential to empower caregivers. Some clinicians mentioned how they trained caregivers on leading and facilitating support groups themselves. A few added that they tried to help caregivers create their own self-help groups. However, they also mentioned that few professionals had the attitude or capacity to work along these principles.

*PCP114, clinician*

*“Parents needs to be included in everything that you do. So that if you're doing some kind of therapy, parents should see, so that they can continue at home. So it shouldn't be you every time doing it. You know, like, we want parents to be empowered so that they can help kids. But this is not what is happening in most cases. It's just like a therapist doing something to the child. And they have, if they need something to be done, the parent should bring the child every time but parents are not being equipped.”*

## Discussion

In this study we explored the meaning of and role that caregiver empowerment may play in developing support services for children with DDs in Ethiopia. In the first theme, we covered socio-economic barriers that undermine caregiver agency – such as living in poverty, or raising a child with DD as a single mother. In the second theme, we covered who caregivers and professionals see as responsible agents for caregiver empowerment and service development. Caregivers, and many professionals saw caregivers as the key instigators, but they also identified that stakeholders like the government, the media, and local and international NGOs should play a more active role in caregiver support. Lastly, in the third theme we discussed how peer support groups for caregivers can help to realise an effective platform for caregiver empowerment.

Caregivers and many professionals saw caregivers at the heart of efforts to support children with DDs. There were examples of resourceful caregivers who initiated change on their own terms, became advocates, and encouraged others to do the same. These operationalisations of empowerment resonate with the literature describing caregiver empowerment as the sense of agency to make effective choices [56], the capacity to balance demands and needs in a family [77], and the ability to change or eliminate potentially stressful events through the application of knowledge and skills [43, 78]. In line with caregiver views, earlier work outlined that caregivers should be given a decision-making position when it comes to developing empowerment interventions [79]. Caregiver and professional participants discussed how caregivers can initiate change for their child with a DD through setting up support groups, advocating for their children at the community level, or saving as a group to open a special school in the future. Evidence suggests that a way in which mothers can be supported in achieving this is through participatory learning programmes targeting health and financial literacy [80].

Our findings also demonstrated the pervasive effects of structural barriers, such as poverty, hindering caregivers from making decisions in the support of their child. Earlier literature has also recorded the association between physical health, undernutrition, and developmental delay in children in Ethiopia [81, 82]. Many caregivers thought that welfare support could help them provide for the basic needs in their family. However, they did not discuss whether or how being able to provide for the basics would be associated with their child’s development.

Participants in our work identified other stakeholder groups that they believed were responsible for initiating financial and social welfare support—as the government, NGOs, and the media. NGO-based participants often talked about the economic empowerment of caregivers, for example, through microfinance initiatives. This was the case even though the literature on microfinance initiatives shows mixed results regarding whether creating economic markets among the poor leads to better economic outcomes or further exploitation [83, 84]. Many NGO representatives used a technical vocabulary regarding poverty eradication: this suggests a professionalisation of everyday activities such as eating and earning a salary, defined as empowerment. Moreover, it means that NGOs can become the decision-makers regarding how a person, or a family should be delivering those everyday activities. The risk of this perspective on poverty eradication is that it recreates power imbalances between donor and beneficiary [85]. Participants mentioned that dependency may occur between local and international providers when funding comes from international resources. This can become a barrier to empowerment of caregivers as tied funding may not use caregivers’ concepts and local resources, but rely heavily on values and concepts from abroad. There were a few examples of caregiver-led initiatives for economic empowerment without external agents being involved. Such a caregiver-centred, decentralised approach could be a way forward in achieving better financial outcomes. However, it also means additional demand on caregivers whose capacity may already be extremely limited.

Many mothers in this study raised their child with a DD as single mothers as their partner left upon knowing about the child’s disability – similar to findings of earlier work [1, 17]. An important component not highlighted in previous studies is the impact of domestic violence. Intimate partner violence exercised by the husband is widespread in Ethiopia [86, 87], as it is globally. Some women caregivers mentioned that they left their husband to avoid abuse. Single mothers in this study who reported having experienced abuse spoke about the additional challenges they experience due to not having a partner, including severe financial difficulties. However, they also talked about their separation from the father as a relief. Experiences of single mothers, and especially those who had experienced violence, point to the need of an intersectional approach to caregiver interventions [1]. Interventions need to consider the economic, physical, and mental well-being of both the caregiver and the child with a DD. Examples to how this approach may be implemented in practice is holistically assessing the physical and mental health of the child and the caregiver; and assessing the living conditions of the family, with special attention to signs of domestic violence.

A potential way forward is bringing caregiver values and priorities to the centre of discussions about empowerment among local and international stakeholders. In Table 3 we summarised possible empowering techniques mentioned by caregiver and professional participants discussed above. Those professional stakeholders who currently have the capacity to develop and implement empowerment interventions could work even more closely together with caregivers and prioritise support for the scale-up of local caregiver initiatives. This also means dedicating resources to bring caregivers to the table – for example, caregivers may need to be paid for their time when taking part in research or the development of new support programmes.

**Table 3:** Empowering techniques mentioned by participants

Ability to shape support systems	A rights-based approach across health, social welfare, and education
	Caregivers are involved in developing support systems for their children
Access to resources	Financial support for caregivers to mitigate poverty
	Social welfare support for caregivers
	Giving access to education to the child with a DD
Access to information	Rights of children with DDs
	Information on what support systems are in place for families
Service provision	Intersectional approach to support single mothers and families experiencing intersecting challenges
	Mental health support
	Physical health support
	Caregiver-mediated support groups and caregiver interventions

## Limitations

In the Ethiopian context, DDs primarily come to clinical attention when delays are moderate to severe; and most people with a formal DD diagnosis are minimally verbal. This study therefore relied on caregiver and professional perspectives only and lacked the voices of a child or a person with disability. The professionals who we recruited were committed to the idea of empowering families. This study is missing the voices of clinicians who have never used caregiver interventions or who believe that such programmes are not the most helpful way of supporting families. Of the caregivers interviewed, all were women and therefore fathers’ voices are missing from the data. We mostly included the voices of those families that have already had contact with health care at least once and had accessed diagnostic services. This is extremely rare in the Ethiopian setting and the voices of those who do not receive any support at all are underrepresented.

Lastly, there were various barriers to reaching and engaging caregivers to discuss findings, including language, culture, infrastructure and the COVID pandemic. The nature of the feedback we received from participants remained on the level of consultation instead of deeper engagement [88].

## Conclusions

Caregivers of children with DDs in Ethiopia are often disempowered through poverty, stigma, and poor access to information and resources that would allow them to make the choices they value in caring for their child. Definitions and understandings of empowerment of caregivers of children with DDs in Ethiopia vary. Caregivers identified needs and expectations from health and social care institutions and spoke about their worries for the future. They then identified what they are already able to do: setting up support groups, leading on initiatives, starting advocacy work. Caregiver empowerment in service development could mean involving caregivers in decisions about support services, matching their vocabulary, and following the agenda set by them addressing their needs.

### Supplementary Information

**Additional file 1. **Full interview guides.

**Additional file 2. **Final codebook.

**Additional file 3. **How the researcher was seen by participants.

**Additional file 4. **The full list of relevant quotes.

**Additional file 5. **COREQ checklist.

## Data Availability

The dataset(s) supporting the conclusions of this article is(are) included within the article (and its additional file(s)).

## Abbreviations

CST: Caregiver Skills Training
CHEW: Community-based health extension workers
DDs: Developmental disabilities
IA: Ikram Ahmed
NGO: Non-governmental organisation
WHO: World Health Organization
ZS: Zsofia Szlamka
