# Supplementary material for: Conceptualising the empowerment of caregivers raising children with developmental disabilities in Ethiopia: a qualitative study
Source: BMC Health Serv Res. 2023 Dec 15;23:1420. doi: 10.1186/s12913-023-10428-4 (PMC10722818; doi:10.1186/s12913-023-10428-4)
Supplement: Supplementary file 1 — Additional file 1. Full interview guides. [file 12913_2023_10428_MOESM1_ESM.docx]

**Additional file 1: Full interview guides**

*Interviews in Amharic will be facilitated by a research assistant and those in English by the UK visiting investigator*

**Topic Guide for Caregivers in Ethiopia**

In this interview I’m interested in hearing about your child who is developing slowly and what you think would be helpful for your family.

**Experiences and needs**

Can you tell me about your last week? What happened to your child? What happened to other family members?

What was the best part of the week? What was the most difficult part of the week? Are these experiences regular?

If you have any concerns about your daily life or your family, who do you talk to? How/why did you choose this person?

Is there someone else you wish you could talk to about your concerns? Would they be able to help? If so, in what way?

Let’s now talk about having a child who is developing slowly. If you have any concerns about your child, who do you talk to? What do you talk about with this person? [neighbours, friends, doctor, teacher, health worker, religious leader]

Is there anyone who you think could help you in some way? In what way could they help?

What would you like to have or provide to your child that you think would help your family?

Can you tell me about things that you find important to provide your child or family with? Is there something you would like to be able to provide but you can not as for now? If so, can you tell me more about this?

Who do you think should know more about children developing slowly? Can you give examples of an organisation or a person?

Do you have any concerns about your child now? If so, what are they?

Do you have any concerns about your child in the future? If so, what are they?

What do you do about these concerns? How do you try to overcome your concerns?

**Experiences with caregiver interventions**

*For those who did not attend CST and have experience with other parents’ groups/interventions*

Have you ever attended a programme specifically for parents who have a child developing slowly? Can you tell me about it? [How did you get to know the programme? Why did you attend? What was your expectation from it? How was your experience?]

What is your experience with meeting parents who have children who develop similarly to your child?

Following the programme you attended, what do you think you would need to overcome the challenges you are facing?

*For those who did not attend CST and have no experience with other parents’ groups/interventions:*

How have you (as an individual, as a family) tried to overcome the challenges you face? What has helped you? What has got in the way?

What do you think could help you to best support your family and child?

Can you tell me about things that you find important to support your child or family with? Is there an area where you would like to offer support but you can not as for now? If so, can you tell me more about this?

Have you heard of anything (from other families, neighbours, friends) that exists in other towns, in Addis Ababa or other countries that you think could help you?

*For those who attended CST:*

You attended a programme for caregivers to help with children who are developing slowly. What did you expect from a programme like CST? Have you previously attended anything similar to this programme?

What is your experience with meeting parents who have children who develop similarly to your child? Do you meet them outside of the CST setting?

Following the programme you attended, what do you think you would need to overcome the challenges you are facing?

**International presence**

You might have met people coming from other countries to work in your town. Have you come across such professionals before? If so, what is your experience in this regard? What do you think about their presence?

**Topic guide: NGO, health extension worker and academic representatives**

**Meeting the needs**

Can you tell me about what your organisation is doing? Can you tell me briefly how you started working in your area of expertise?

How is your experience working with these families and these children? What do they tell you about?

In your experience, what do families raising a slowly developing child need? How did you get to know this?

What do you think they would need to better help their children?

What do you think you would need to provide care even better?

How do you think these needs can be met? How should this be done in practice?

Who do you think is responsible for making sure these happen? Who should initiate these practices?

How do you see your organisation’s role in helping these families? What could be done from your organisation’s side? What should happen so that your organisation can achieve these?

Do you work with lower income groups? What is your experience working with them? What kind of challenges are they facing?

I heard from some organisations that it is mainly mothers that they meet. Do you meet father as well in your work?

I also heard that sometimes fathers or mothers leave the family when they get to know that their child is developing slowly. Did you come across such a situation before? What is the single parent’s experience?

How is your experience with working with different stakeholders? How is it to work with government? How is it to work with funding agencies?

**Empowerment**

In a perfect world, what would be available for these families?

When talking to different organisations, the concept of empowerment of families came up on occasion. However, what I found was that different people mean different things by empowerment. What does empowerment mean to you (in the context of working with families with a child with DD)? What would it mean for a family to be empowered?

What do you think would be a good outcome of empowerment? What would you consider as successful? What would you consider as unsuccessful?

To what extent is empowerment a goal in your work?

Are there any techniques that work well to achieve these goals? Are there techniques that do not work well? How do you know?

What does inclusivity mean to you? How do you think society approaches inclusion at this point?

What would be the end goal?

Did you come across advocacy in your field? What is your experience with advocacy?

How do you see the role of research and generating evidence in this field?

Have you come across health innovations or financial innovations in the field, such as crowdfunding platforms or social impact bonds?

**Experiences with caregiver interventions**

Are you training parents in your work or organisation? Can you tell me about your experience with them?

How do you see the role of training parents overall in terms of providing care and services?

*For those working with caregiver programmes:*

Let’s talk about the intervention you are working with. How did you choose this intervention?

How would you summarise the key goal this intervention is hoping to achieve?

What are your experiences with supporting caregivers and families attend the intervention? Are there any key challenges of engaging caregivers?

How do you think caregivers should be approached regarding the participation in the intervention? Do you have any practices that worked well in this regard?

What do you think about the participation of caregivers and families from lower socio-economic backgrounds?

Did you find any approaches or practices that work well to engage caregivers from a variety of backgrounds? Did you find approaches that didn’t work well in this regard?

What do you think about paying caregivers to attend the intervention?

**Training and teaching (in case if the participant has relevant experience)**

In the intervention you are working with, there may be concepts introduced that are unfamiliar to the target audience. Have you come across such a situation before? If so, what was your experience?

Were there any concepts that caregivers found difficult to understand? Did you find any solutions to this? Did you find any techniques that worked particularly well explaining new concepts to caregivers? Did you find any techniques that proved to be unhelpful?

Were there any teaching methods or strategies that the caregivers found difficult to understand? Did you find any solutions to this?

Do you have any good practices of how to help caregivers attend to the programme?

In your experience, was there something caregivers were paying close attention to? Can you give examples?

In your experience, was there something caregivers didn’t find relevant? Can you give examples?

**Supporting intervention providers and caregivers**

Can you tell me your experience about working together with caregivers?

What is the role of those working in your organisation?

What is the role of the caregiver? What is the caregiver’s contribution?

How do caregivers get what they need from your programme? What interferes with that?

Do you have any good practices of supporting that those working in your organisation and caregivers remain involved in the intervention?

Can you tell me about any stakeholder groups you are working with regularly or on occasion? What is it like to work with different professionals and different groups?

Is there something that works particularly well with any of these groups?

Is there something you find challenging when working with them?

**Pricing of interventions and stakeholder groups**

Let’s now talk about how interventions are financed. Some people argue such programmes should be free while others state participants should pay a fee to attend. What do you think about this? Can you explain why?

How do you think the price may relate to how the intervention works?

**Responsibilities**

You might have met professionals coming from other countries to work on programmes for families, for example from United Nations or international NGOs, international researchers. Have you come across such professionals before? If so, what is your experience in this regard? What do you think their role is in the field of developmental disorders?

**Topic guide: Local municipality/authority**

**Meeting the needs**

What type of activities do you know of for people with disabilities in this district? How do you know about these activities? How are they relating to your sector and profession?

*For those who have more experience with interventions:*

Let’s talk about interventions in general. How do you get to know existing health programmes in your district or in neighbouring districts? How do you know existing programmes of empowerment in your district or in neighbouring districts?

What would you like to know about a programme before you decide whether to give your support? How do you go about choosing interventions to support?

Once a programme is supported, how much do you remain involved in its development?

Which stakeholder groups would you like to see in the development of these programmes?

What are your expectations from these interventions?

What do you think about getting communities from a variety of backgrounds attend these programmes?

What do you think about paying participants to attend such interventions?

What questions would you ask once the intervention programme is finished?

Have you come across the WHO’s Caregivers Skills Training before? If so, how did you get to know CST? What was your experience?

Are there similar programmes you got to know targeting caregivers of children with disabilities?

How was the decision made to work towards the needs of such families? To what extent do you think CST helps meeting the community’s needs? How do you and your organisation know?

**Empowerment**

What does empowerment mean in your understanding? What do you think the goal of empowerment is?

What are the means of empowerment? Are there techniques that do not work well?

How do you think this intervention is achieving these goals?

In case of some of the interventions, I came across partners such as the United Nations and international NGOs as well as researchers from other countries coming to Ethiopia and working on it. What do you think about this? What is your experience with working with many stakeholder groups? How do you find dealing with many projects at the same time?

**Pricing of interventions and stakeholder groups**

Let’s now talk about how interventions are financed. Some people argue such programmes should be free while others state participants should pay a fee to attend. What do you think about this? Can you explain why?

How do you think the price may relate to how the intervention works?

Can you tell me about any stakeholder groups you are working with regularly or on occasion? What is it like to work with different professionals and different groups?

Is there something that works particularly well with any of these groups?

Is there something you find challenging when working with them?
