# Supplementary material for: Conceptualising the empowerment of caregivers raising children with developmental disabilities in Ethiopia: a qualitative study
Source: BMC Health Serv Res. 2023 Dec 15;23:1420. doi: 10.1186/s12913-023-10428-4 (PMC10722818; doi:10.1186/s12913-023-10428-4)
Supplement: Supplementary file 2 — Additional file 2. Final codebook. [file 12913_2023_10428_MOESM2_ESM.docx]

**Additional file 2: Final codebook**

Codes

| Name | Description |
| --- | --- |
| Building an infrastructure meeting the needs of families with NDDs |  |
| Appreciating progress in the context of remaining gaps in services |  |
| Existing social support |  |
| Experiences with CBR | CBR workers are very good at transferring knowledge to parents, how to find the right challenge for the child, no quality control of CBR workers, officers have to be trained of sign language |
| CBR workers are very good at tranferring skills to parents |  |
| No quality control for CBR workers in NGOs |  |
| There has been progress from a gender perspective |  |
| Caregiver interventions | what happens after the baseline service, caregiver interventions are crucial in Ethiopia, but with specialist services, how is it to train illiterate caregivers, in some places caregiver training is only related to home-based rehabilitation, but professionals usually start doing it intuitively |
| Caregiver training can take place hand in hand with poverty eradication |  |
| Caregivers expect the supporting organisation to take over the burden |  |
| Challenges with interventions | no child care facilities, need to compensate parents for their time, they may bring individual problems, caregivers expect a cure, they may not be able to generalise skills from the hospital to the home, how caregivers get to know about the intervention, oftentimes there's a long waiting list, problems with attendance |
| How do we know if individualised techniques are effective |  |
| When expectations do not meet the reality |  |
| Goal of caregiver interventions | so that caregivers know how to support their children, they understand them, they should be involved in the therapy of their child, materials for the family if they can't afford specialists, psychological support for caregivers |
| Stakeholder experiences with interventions | it can get boring to keep teaching their children |
| Developing interventions |  |
| Experiences with engaging with caregivers |  |
| How to adapt interventions to different cultures within Ethiopia |  |
| Not all children are identified and receive support |  |
| Professional caregivers experience a time constraint |  |
| Training often takes place in informal ways |  |
| Resources needed to develop services |  |
| Better referral system |  |
| Interventions and techniques |  |
| Training outside of Addis |  |
| Location |  |
| regional differences in services |  |
| urban or rural |  |
| More funding | support from the hospital |
| Transportation costs for parents |  |
| More human resources |  |
| More information to parents is needed |  |
| More professionals and specialists trained on NDDs |  |
| Some of the children will need life-long care |  |
| The personal motivation driving change |  |
| The process in public health | responsibilities of health workers, supporting children from orphanages, no medication supply, inpatient services |
| Health extension workers as the entry point to support | work takes place is there is a donor, urban or rural, referral system, HEW and mental health, education and training, competency, transfer needs, no medication, lack of skills, income barriers, higher salary, high turnover |
| How the Women Development Army works |  |
| Why the WDA is only including women |  |
| Women are not paid to take part in the WDA |  |
| women do not participate in the groups because they have to work |  |
| How to identify children with NDDs | and how to identify the poorest of the poor |
| How to inform caregivers and raise awareness |  |
| Perception of HEWs |  |
| Preventing illness in the community |  |
| Knowing what support is needed and not being able to provide it |  |
| The grassroot and policy gap | it happens with inclusion too |
| A dependency syndrome may evolve between the provider and beneficiaries |  |
| Relying on international funding and materials create dependency |  |
| Bureaucratic barriers and corruption | development and funding happens through connections |
| Communication across stakeholders is difficult | like overlap of mandates |
| Participatory approach towards caregivers |  |
| Technocrat language of NGOs |  |
| Cross-sectoral work is needed | there might also be competition that goes against collaboration, teachers and health professionals should work together, developing access to services |
| Challenges of cross-sectoral work |  |
| Examples of collaboration |  |
| Expectations and experiences with how the government handles NDDs | they bring specialists from abroad, should create jobs for assistants for DD, the ministries should take DDs more seriously, when leadership changes the work may stop, |
| getting government buy in |  |
| NGOs think they do the work of the government |  |
| The government should put protocols in place but not alone |  |
| Experiences with community involvement |  |
| NGO experiences with NDDs | from awareness to income generation, the results are good when devices or simples materials have to be provided, autism centres are costly, community expectations of NGOs, international connections can help facilitate the start of an NGO |
| Experiences of donor organisations and funding | how to collaborate with donors |
| NGOs may refer NDDs to autism centres but there are not many of them |  |
| Plans to expand services | for example in other regions or by having more space |
| Relying on volunteers |  |
| What beneficiaries expect from NGOs |  |
| No professional boundaries set | but having someone available will at least achieve something, if a professional doesn't know what the problem is, they will mistreat it, no relevant training for families and professionals |
| Organisations may have a very different viewpoint on working with NDDs |  |
| Research and evidence | it seems to be a vicious circle that in order for the UN to go and do work around NDDs, data are needed; but for that a stronger government and health system is needed, or a strong research background and that would often come with international funding |
| Evidence is needed to reach actual commitment of decision-making bodies |  |
| The UN and NDDs | No work with grassroots, no overwhelmed officials, role of WHO |
| Nominal or actual commitment to NDDs |  |
| Priority setting in a UN system and how NDDs may or may not fit in |  |
| Whether to get nationwide data on NDDs if there are no services |  |
| Weak child protection system in Ethiopia |  |
| How priorities are set | DDs are often not priorities, accidents, socio-economic problems, malnutrition, mortality and internally displaced people are priorities |
| NDDs are not priorities | there is a general focus on disabilities, no one takes responsibility, service provision and referral system are not built, what are the available public services for DDs, NGOs don't seem to work much in this area either, no one takes responsibility |
| Challenges with the referral system |  |
| Focus on physical disabilities or disabilities in general |  |
| NDDs in Ethiopia from a policy perspective |  |
| Weak mental health system |  |
| Empowerment, inclusion, and advocacy to promote service development |  |
| Advocacy as a driver of service development | There is not enoug advocacy and collaboration needs to happen |
| Challenges of advocacy |  |
| Means to advocate |  |
| The overlap of empowerment, inclusion and advocacy |  |
| Having materials and resources is the base for empowerment, inclusion and advocacy |  |
| Inclusion and empowerment based on charity or rights |  |
| What to achieve with advocacy | Achieve awareness raising: among government officials, parents, people with disabilities, among community members; awareness leads to income and decreased stigma, people's feelings may be hurt and there has already been considerable progress made |
| Advocacy should be strategic |  |
| Break through the gap between physical and intellectual disabilities |  |
| Founding a parent association |  |
| Needs across disabilities might be different for advocacy |  |
| Persons with disabilities should also be involved, not just caregivers |  |
| The goal is to raise awareness | among government officials, parents, people with disabilities, among community members; awareness leads to income and decreased stigma, people's feelings may be hurt and there has already been considerable progress made |
| There is favouritism towards autism |  |
| Universal design |  |
| Who should know more about NDDs and therefore support service development |  |
| Caregivers leading initiatives |  |
| Advocacy as a question of coming out |  |
| Caregivers initiating schools |  |
| How to manage the school |  |
| It is not possible to help everyone at once |  |
| Learning how to teach by experience |  |
| Other caregivers contribute with materials so that the school improves |  |
| Training caregivers and its challenges |  |
| Having more parents together means more power to advocacy |  |
| In some cases there is no networking among parents yet |  |
| Initiatives for a parents association |  |
| Support groups | health professionals often have a facilitating role, caregivers discuss stress, support with house rent or finance |
| Diversity of NDDs is a barrier to support groups |  |
| Parent communication supports their learning |  |
| Time constraint is a barrier to support groups |  |
| The role of female support groups |  |
| Women as productive workers |  |
| Worries and wishes about the child | particularly about the future |
| Economic empowerment through poverty eradication | it can create advocacy and speaking up for thier rights |
| Economic empowerment should be the first step |  |
| Good commitment achieved to repay loans |  |
| Large scale programmes exist to support poverty eradication |  |
| Persons with disabilities are rational |  |
| The narrative of us and them |  |
| Experiences with inclusion | it means specialist services, can be achieved through awareness, decreases stigma, making other areas of life disability inclusive, there's a two-way approach, a UN approach and a NGO approach, it needs the understanding of child development, inclusion should happen on the ground level |
| Education needs |  |
| Caregiver needs in terms of education | conflict with teachers because of misconceptions about DDs, few professionals in school, pushing too much, needing schools to then be able to work, the child might be stressed in school, this can impact toilet training |
| Caregivers have to be involved to generalise skills |  |
| Challenges in education | turnover of staff |
| How to get a licence from the government |  |
| Licence for institutions is given by showing the economic impact |  |
| Need for trained teachers |  |
| Prioritisation in giving access to education |  |
| Decentralise knowledge so that it is affordable for everyone |  |
| Education is a means to eradicate poverty for those living with a disability |  |
| Experiences of special education teachers | stigma on special needs departments, NGOs provide training, lack of commitment after training, how to overcome bullying, expert versus intuitive knowledge |
| Experiences with private autism schools | work for profit, teaching plan, difficulties; how could this system be replicated to government schools as well? |
| How to scale the private system to the public system |  |
| The cost of private schools |  |
| Experiences with special education |  |
| Transferring from special needs to government schools |  |
| What happens when the child in special education grows up |  |
| What happens with the child after school |  |
| How to advise policy on inclusion |  |
| How to be disability-targeted |  |
| Inclusive education | teachers lack skills to do inclusive education and they don't have resources, teachers should be sensitised, comparing children is not working, consistency is essential |
| Experiences with different forms of inclusion in schools | what happens when inclusion takes place not because of a conscious decision, bit because there is no money for further classes |
| Children without NDDs also learn about inclusion |  |
| Labelling children without diagnosis |  |
| Wishing for separated classes |  |
| How to find teachers and support workers to help children with NDDs |  |
| Caregivers may be able to get trained and help others |  |
| Support workers are all females |  |
| Teacher training often takes place through informal training |  |
| How to include in practice | inclusive, integrated or segregated; inclusive classroom may not provide for all needs |
| How to build structured inclusive education |  |
| Showing the school environment to neighbours to raise awareness |  |
| Strenght perspective for children |  |
| Providing materials |  |
| Some teachers are afraid of inclusion |  |
| What is the goal of inclusive education | to make the child a useful member of society |
| Lacking inclusion leads to hiding the problem |  |
| Some caregivers of children without NDDs are afraid of inclusion |  |
| The professional view on empowerment |  |
| Empowerment as a skill | it has lots of meanings; it depends on the profession |
| Empowerment is education and resources for persons with disabilities |  |
| Empowerment means access to families' rights |  |
| Examples |  |
| Twin track approach |  |
| Women Development Army | voluntary, gengdered, official in the kebele, engaging the community |
| Having skills and information to manage the child |  |
| Independence and being organised |  |
| Persons with disabilities claim their rights |  |
| Self-worth and self-defence against stigma |  |
| Empowerment as only a nominal commitment |  |
| Empowerment in education should be against corporal punishment |  |
| Empowerment on the level of organisations and civil voices | no local capacity and no fundraising experience, empowering through office equipment and human resources, there is a need for an individual who drives change |
| Empowerment outcomes | it can lead to people doing voluntary work, when parents are empowered, it will dirctly affect the children, parents form networks, they feel a school or an organisation is theirs |
| Having data drives empowerment |  |
| How to measure empowerment |  |
| Mainstreaming as a tool of empowerment | Mainstreaming to women and poverty for example, but mainstreaming is not enough |
| When empowerment is unsuccessful | and if there is no empowerment component |
| Drawbacks of empowerment | by being the target of empowerment, not being equal is emphasised, why do they need special treatment |
| What does it mean not to be empowered |  |
| Good quotes |  |
| Advocacy |  |
| Beliefs |  |
| Awareness |  |
| Caregiver experiences |  |
| Caregiver interventions |  |
| Empowerment |  |
| Religion |  |
| Gender |  |
| Grassroot and policy gap |  |
| Health system |  |
| Inclusion |  |
| International presence |  |
| Policy |  |
| Poverty |  |
| Leaving the child for NGOs |  |
| Outlier cases |  |
| A dependency syndrome may evolve between the NGO and beneficiaries |  |
| International organisations may reinforce existing problems |  |
| Advocacy |  |
| Appreciating progress in the context of remaining gaps in services |  |
| Existing social support |  |
| Experiences with CBR | CBR workers are very good at transferring knowledge to parents, how to find the right challenge for the child, no quality control of CBR workers, officers have to be trained of sign language |
| CBR workers are very good at tranferring skills to parents |  |
| No quality control for CBR workers in NGOs |  |
| There has been progress from a gender perspective |  |
| Caregivers who killed their child with a disability |  |
| Data on prevalence |  |
| Education and school |  |
| Empowerment as a nominal commitment from a UN perspective |  |
| Father as the caregiver |  |
| Power in the context of research in Ethiopia |  |
| How the researcher is seen by participants |  |
| Perceptions of the work in and outside of Ethiopia | internationals are needed because there is no local capacity, funding and there are many ethnic problems, it is an opportunity to upstream an agenda, the view of the global north |
| Beliefs about what may or may not be available abroad |  |
| Caregivers living outside of Ethiopia |  |
| Expectations from the researcher's organisation |  |
| Experience of working in Ethiopia as an international |  |
| How locals encounter internationals |  |
| Internationals initiating an organisation based on what they saw in Ethiopia |  |
| These initiatives may reinforce existing problems |  |
| Perceptions of expats about receiving support |  |
| Receiving materials from internationals |  |
| Contextual and cultural barriers to using materials |  |
| Language barrier to using materials in Amharic |  |
| What internationals think locals think or experience |  |
| Volunteers who want to help the poor and needy |  |
| The experiences and challenges of mothers |  |
| Caregiving from a gendered perspective |  |
| Father as the main caregiver |  |
| The father's position in the family |  |
| It is not possible to share parenting challenges with the father |  |
| The father living abroad |  |
| What is success for women |  |
| Experiences of single mothers |  |
| Not sharing problems with anyone |  |
| The question of marriage | whether to re-marry, the advantage of being separated, feeling ashamed because of being single, feeling angry about the father who left, relationship with the ex-husband |
| Experiencing violence, abuse or discrimination because of gender |  |
| Appreciating children of both sexes |  |
| Caregivers who killed their child with a disability |  |
| Corporal punishment of the child |  |
| Domestic violence and violence against women |  |
| Experiences of women and girls with disability |  |
| How professionals unconsciously add to gender discrimination |  |
| If the woman speaks bad of the husband, no one will help |  |
| Finding support when raising a child with an NDD |  |
| Caregiver physical and mental health | hopelessness, what brings joy in life, need help to grow to maximum limits, it depends on SES, coping strategies |
| Coping as a caregiver | vocational training, they only receive diagnosis, pay for service in the lack of available ones, learn skills, speech therapy, how to generalise from school to home, wanting the child to be normal, address the basics first |
| Asking for support and speaking up for the family |  |
| Experiences with receiving support |  |
| Failures in receiving support lead not to ask for more |  |
| Relationship with other parents |  |
| Relationships within the family | how other family members view the child with an NDD |
| Religion |  |
| as a barrier to development |  |
| as a coping strategy |  |
| The journey of finding help | how the caregiver realised that there was a developmental delay, wanting spiritual help |
| Who is a support person to the caregiver | father is often not a support person, mothers with the same issues are |
| Raising a child with an NDD in day-to-day life | relocate, learn about NDDs, when the child i shappy, there is no way out of it, receiving support, everyday routine, managing unwanted behaviours |
| Daily routine for the family |  |
| Challenges of everyday life |  |
| What is good in everyday life |  |
| Managing comorbid conditions of the child |  |
| The caregiver may only focus on the child with NDD |  |
| How people think about NDDs | no proper diagnosis and identification in NGOs, |
| Beliefs and attitudes regarding NDDs | if the child is abroad, then can get medicine and recover, because the child looks normal, the mother might not get time off work, beliefs differ per location, beliefs about work, NDDs are the same everywhere, how we approach them differs |
| Beliefs about what caregiving should look like |  |
| Assumptions about families raising children with NDDs |  |
| Beliefs about what certain support systems could achieve |  |
| Religion setting the goals for an organisation about NDDs |  |
| How a child with an NDD is able or is not able to live |  |
| People are afraid of NDDs |  |
| Persons with NDDs can not be equal |  |
| Questioning certain beliefs |  |
| The gap between physical and intellectual disabilities | no easy solution, because intellectual disability is invisible, because health workers don't know how to address intellectual, an organisation may not be intentional about working with NDDs - they come across mental disorders when working with disability generally speaking |
| Why a child has an NDD |  |
| Beliefs about pregancy and disability |  |
| It takes time for caregivers to understand NDDs |  |
| Mothers may have a conflict with the family about the child |  |
| Stigma and awareness around NDDs | self-isolate not to face stigma, no caregiver awareness, keep children at home, stigma leads to the fact that there's not many projects around NDDs |
| No awareness of NDDs | even among professionals of HEWs |
| Unreliable national data on NDDs |  |
| Self isolation because of stigma |  |
| The location influences stigma | there are locations where may not be stigma |
| When the surroundings accept NDDs |  |
| The impact of poverty on the quality of life of families with NDDs |  |
| Difficulty of making a living | having schools available would allow them to work, they sometimes receive money from male family members |
| Access to job opportunities |  |
| Caregivers need a day care centre so they can work |  |
| Caregivers can not provide for basic needs |  |
| Caregivers can not afford resources |  |
| Family arrangements at home |  |
| Caregivers wanting to leave the child | send the child to the US, or leaving the child with an organisation |
| Money as an enabler |  |
| Illiteracy and lack of education |  |
| Challenges with understanding the support system |  |
| No health education |  |
| The family has to prioritise | it is a luxury for families to pay for the therapy of a member who is not otherwise contributing |
| The relation of disability and poverty |  |
