# Supplementary material for: Conceptualising the empowerment of caregivers raising children with developmental disabilities in Ethiopia: a qualitative study
Source: BMC Health Serv Res. 2023 Dec 15;23:1420. doi: 10.1186/s12913-023-10428-4 (PMC10722818; doi:10.1186/s12913-023-10428-4)
Supplement: Supplementary file 3 — Additional file 3. How the researcher was seen by participants. [file 12913_2023_10428_MOESM3_ESM.docx]

**Additional file 3: How the researcher was seen by participants**

**Name:** How the researcher is seen by participants

<Files\\PCP226> - § 2 references coded [1.92% Coverage]

Reference 1 - 1.26% Coverage

Take away. We don't get the credits. We don't get the I don’t know financial support. We don't get the privilege to visit or to share other you know, places, your knowledge. For example, you will go and talk about this. You will gain from the audience, right? Because you're talking with them and you are sharing experiences. You will gain experience, right, when you go there, talk with others…

Zs: Even the fact that I'm here, it gives experience.

PCP226: Experience. Yeah. But if I don't get that chance, I don't get anything. All I do is just give, give, give, give, give, talk, talk, talk, talk, talk, talk.

Reference 2 - 0.66% Coverage

I can tell you this the reason, for many that they're happy is because you come from another world. And again, the mindset here is that they feel you are full of knowledge. You are the angel, you know, you are. And then they feel that they know nothing. Yeah. See, how did it How did this kind of thinking come? I don't know.

<Files\\PCP311> - § 1 reference coded [0.53% Coverage]

Reference 1 - 0.53% Coverage

what I ask you is that, I am not the only person you are going to interview. Whenever you go and interview, I wonder if you can ask if people really understand inclusive education, one.

<Files\\PCP515> - § 1 reference coded [5.63% Coverage]

Reference 1 - 5.63% Coverage

I think this is a very important initiatives where it helps us to see the reality very much clearly because you're going around and discussing these different papers or different knowledge, experiences, skills and so, that will finally give us the clear picture of where we are and also YOU will come up with some kind of recommendation as an expert and as someone who was revised and underwent various kinds of exploration. So the recommendations which are going to come from your journey will be very much helpful in terms of addressing the need of these people. They're not small number. So many knowledge and experience is required on all these regards, so we'll be waiting for the final report of your work. So that we can consider it and consider people like you on how we should go forward towards satisfying all these concerns. So, my final remarks will be just thanks for you for this effort because we know that it will be helpful because there are limitations in understanding the existence of the solution and how much the response is, there are responses which are scattered as you might have understood, but bringing this together for this opponent will be very much important, and I think that will be that will come from your side and, and furthermore, because you're from the West where the services and work around these special issues is more advanced. So I think we will get more wisdom and advice through the work you're doing. So it's nice to have time with you is to wait for the final product and more discussion.
