# Supplementary material for: Conceptualising the empowerment of caregivers raising children with developmental disabilities in Ethiopia: a qualitative study
Source: BMC Health Serv Res. 2023 Dec 15;23:1420. doi: 10.1186/s12913-023-10428-4 (PMC10722818; doi:10.1186/s12913-023-10428-4)
Supplement: Supplementary file 4 — Additional file 4. The full list of relevant quotes. [file 12913_2023_10428_MOESM4_ESM.docx]

**Additional file 4: The full list of relevant quotes**

| *PCP1455, caregiver, Addis Ababa* | *PCP1455, caregiver, Addis Ababa*  *“I took him to the hospital and they tested his blood and checked his ears. I couldn't believe them when they told me that he is autistic. I didn't know that [autistic] children looked [normal] like him. She taught me about it in the appointment days, and I learned about it after a long time. She also helped me to get the training. He [my husband] was not happy with my decision to get the training, but I convinced him about it and I am getting the training until now.”* |
| --- | --- |
| *PCP0935, caregiver* | *“She is nine years old, and it has been six years since she started going to school. She went to nursery school when she was three years and five months old, but she can't read and write yet. She speaks very well, and she has a healthy eating habit. She also looks normal physically. I always feel upset when I see what other kids have written on their exercise books or have scored in their tests. I thought that her situation could improve in a year or two. I didn't think of it as an illness at first, and I just took her to a tsebel (holy water) place. It is the school that encouraged us [the family] to take her to the hospital.”* |
| *PCP1315, caregiver* | *“I have a close relationship with her [daughter]. She is not yet six, but she takes care of her brother [with an NDD]. She treats him [with love]. Parents get upset when he attacks their children because they think that he attacked them on purpose. She tells them that he doesn't speak, and he didn't attack their kids on purpose. She helps me with things like this. He also attacks her in the house or when they play outside the house. She tells me that he didn't hit her intentionally and calms me down when I get upset. She tells me that he didn’t hurt her.”* |
| *PCP1408, caregiver* | *“I always give priority to my son. I attend any wedding or other social events on weekends. I don't leave him at home and go away somewhere because I fear that something wrong could happen to him in my absence. I take him with me when I visit my family.”* |
| *PCP1455, caregiver, Addis Ababa* | *“I took him to the hospital and they tested his blood and checked his ears. I couldn't believe them when they told me that he is autistic. I didn't know that [autistic] children looked [normal] like him. She taught me about it in the appointment days, and I learned about it after a long time. She also helped me to get the training. He [my husband] was not happy with my decision to get the training, but I convinced him about it and I am getting the training until now.”* |
| *PCP0935, caregiver* | *“She is nine years old, and it has been six years since she started going to school. She went to nursery school when she was three years and five months old, but she can't read and write yet. She speaks very well, and she has a healthy eating habit. She also looks normal physically. I always feel upset when I see what other kids have written on their exercise books or have scored in their tests. I thought that her situation could improve in a year or two. I didn't think of it as an illness at first, and I just took her to a tsebel (holy water) place. It is the school that encouraged us [the family] to take her to the hospital.”* |
| *PCP1315, caregiver* | *“I have a close relationship with her [my daughter]. She is not yet six, but she takes care of her brother [with an NDD]. She treats him [with love]. Parents get upset when he attacks their children because they think that he attacked them on purpose. She tells them that he doesn't speak, and he didn't attack their kids on purpose. She helps me with things like this. He also attacks her in the house or when they play outside the house. She tells me that he didn't hit her intentionally and calms me down when I get upset. She tells me that he didn’t hurt her.”* |
| *PCP312, school leader* | *“I would tell them use spiritual intervention, it is good on mental health, it’s very important, we shouldn’t ignore that, but I tell them…have time for the spiritual things…but don’t forget also having time to practice, to teach the child.”* |
| *PCP0313, caregiver, Addis Ababa* | *“I always come to this hospital. It is like my home. I come to the mental health and ophthalmology departments, and the doctors give me advice. And my neighbours tell me to take him to people who recite the Holy Quran and to the tsebel (holy water place).”* |
| *PCP0232, caregiver* | *“It would be good for anyone to do work and be independent of other people. You can't leave this kind of child with other people [in the house] and go to work. As for me, I should take him with me if I should go to work. I know many people who don't have any money and can't go to work because they have children with similar problems as mine. I think it would be good if we [people who have children developing slowly] could help one another.”* |
| *PCP1139, HEW, Butajira* | *“People who have economic problems ask us for advice about what they should do to take care of their children. If we tell them to take their children to Addis Ababa for treatment, they will just say "ok" -- and they will not implement our advice. They will just keep their children locked in the house.”* |
| *PCP0232, caregiver* | *“I have relatives in the U.S. I wish they could take him there, and he would have a better life. I don't want anything else.”* |
| *PCP1028, caregiver, Butajira* | *“I went to Addis Ababa, and the hospital told me that the medication is out of stock. Then I went to the municipality pharmacy, and they asked me to show them my certificate for free medical treatment. I didn't have a certificate. I finally found the medication from the hospital…I bought medication that is only enough for two months because I didn't have money to buy medication that is enough for four months*…*She [the doctor] told me that it is the woreda administration that gives a certificate for free medical treatment [to poor people] … She didn't understand why I couldn't buy enough medication [for four months].”* |
| PCP1106, HEW, Butajira | *“Aid organizations and foreigners help them when they come here. Some of their needs become fulfilled at that time. The can at least eat food for one or two days. On our [health extension workers] part, we give them priority when some aid comes through the kebele administration. Our kebele [district] has provided about nine to ten cell phones to pregnant women who are very poor and can't afford to buy the same. So I think that aid organizations should give priority to these people.”* |
| *PCP226, caregiver and school leader* | *“When it comes this kind of disorder with poverty and ignorance [a lack of awareness of NDDs], I cannot imagine what kind of life they can lead…If they don't read the experiences of others, if they don't know how to search about the issue, they think they're the only one who is experiencing this and they don't know anything about it, can you imagine what it means?...But I'm able to, I give help. How do I give help, because I'm in a better situation. I can take my child anywhere I want. I don’t have a transportation problem. So, and also I can read, I can search, I can use my internet, I can see what people are doing. But for those mothers who can not do that, they can't take their children out. They cannot buy things for them, support, some of them, they can't even feed them. They cannot change their clothes.”* |
| *PCP0313, caregiver, Addis Ababa* | *I can't carry him and work. He can walk, but he asks me to carry him on my back after he walks for some time...You can't leave him with anyone else. That is why I am not working and going outside the house to meet with people.* |
| *PCP117, clinician, Addis Ababa* | *“Sometimes they say they want to leave the child with NGOs. They can ask us to give their children to an NGO, I have no money, I have no social support, they want to give away the child…They are crying, they cry, and once they can put the child and they need to go home, I have no power, no money to control the children, to give or to feed the children so the government can take the child, I have no money option to deal with this child, and because the child has some problem, she has the problem and she wants to leave the child for the government. The mother can put here and she can disappear, she came to get the medication or the treatment, but she can put the child here and she disappeared, they see it as the government. I have no money to support the child, so they say it’s the government’s responsibility to take the child. And they can put here and they go away.”* |
| *PCP1028, caregiver, Butajira* | *“They [the government] deported us [from an Arab country]. I didn't have any property. An illegal migrant will not buy a property. My husband didn't come back [to Butajira], and he never called us after that time. He didn't want to have a child in the first place because he didn't have money…I didn't want to be pregnant, and abortion is Haram [forbidden by religion]. They [my family] didn't say anything, but I feel ashamed to take her to my parent's house.”* |
| *PCP111, clinician, Addis Ababa* | *“So their husband usually leaves them when they know the child has some kind of problem. The fathers, they might blame the mother…the problem is in your family… It's common to hear such stories.”* |
| *PCP414, caregiver, Addis Ababa* | *“It’s usually not the father and sometimes, I have a lot of miscommunication with a lot of the teachers…sometimes you feel like you're venturing into new territory for providers…you know, if you come and you're single mum, things are challenging. Maybe there's more empathy on the part of the providers… if you come and you're a little bit more aggressive, as a father, you put your foot down and there's an issue, that can cause conflict.”* |
| *PCP1408, caregiver* | *“I always give priority to my son. I attend any wedding or other social events on weekends. I don't leave him at home and go away somewhere because I fear that something wrong could happen to him in my absence. I take him with me when I visit my family.”* |
| *PCP1408, caregiver* | *“I think of when I and my son will be able to understand each other like his friends and their families and feel stressed about that. I think of where and with whom he will live when I die and feel stressed about that.”* |
| *PCP0935, caregiver* | *“God knows about what will happen to her in the future. People have different kinds of gifts. I convince myself that God has given her another gift and comfort myself with that. However, I still can't cope with the fact that she is unable to learn.”* |
| *PCP226, caregiver and advocate* | *“We were in the [same] boat…for some of them…I was kind of a brave mother to come out and say this….What does she have to tell us? Why is she coming out, telling us that her son has some difficulties, so it was you know a mixed feeling.”* |
| *PCP1028, caregiver, Butajira* | *“I am illiterate…I didn't see the doctors at the appointed time because I couldn't get a [shared] taxi on Friday. Friday is a market day in our area. I wanted to go to the hospital on Saturday or Sunday, and the doctor told me that the hospital is not open on weekends. I went there on Tuesday, and they asked me why I was late…They helped me when I cried and told them that I came from a faraway area, and, can't spend a day [in Addis Ababa].”* |
| *PCP0051, caregiver* | *“There is this woman who isn't related to me by blood. She is her [a neighbour’s] aunt. We know each other by phone, and we are family now. I have decided to talk to her about the things I don’t even dare to talk to my priest.”* |
| *PCP1003, HEW* | *“People don't have the same status in life. Some people are poor and others are rich. Children of rich people have better opportunities than children of poor people. So I think that it would be good to empower families to help the children.”* |
| *PCP221, NGO representative* | *“In the headquarters our biggest challenge is parents I can say. These parents come desperately initially, and they leave all the burden on us, they consider us to do everything for them, after comparting them, they realise they’re going to take the burden, they’re going to share with us the burden...”* |
| *PCP119, clinician* | *“I think the training was formed based on the Western society. So, our patient or our society's relationship with a physician is like, give me the medication and I want to like immediate relief. Otherwise they don’t want to give them like, any home assignment or something like that they have to do it by themselves. That's the problem. Okay, even those who are trained, sometimes come to our clinic, like as a new as a new caregiver and ask, what can I do? What can, what kind of solution I can give for my child for this problem? They are already trained.”* |
| *PCP115, clinician* | *“Just because he brings the bread home, he thinks it has nothing to do with problems happening in the family…the additional issue there is the economics actually, especially the mother's capacity… when the woman is totally dependent on the man, it is very difficult to you know, to get things done the way you want it, because you have to get the agreement from the Lord. The father. There, that is a big issue.”* |
| *PCP226, NGO representative* | *“I'm very close with my parents. And I see what's needed and they talk to me individually. I consult them, I advise them. So I know what's in that mother. I also diagnose children. I do assessments. So when I do that, I work with, you know, closely with the parents, so I know what's missing and what they have, you know, so I try to bring parents together and learn one from another.”* |
| *PCP220, NGO representative, Addis Ababa* | *“In most cases, plans do not consider persons with disabilities, what we say is you have to be disability-targeted. If you're planning something, you have to intentionally plan to get the person with disability, or persons with disabilities. What is funny, in most cases I used to meet persons from gender departments, from different organisations. What do you work? We work for the well-being of women workers and something to respect their right, if there is any kind of rights violation. I listen to them: how do you include women with disabilities? No, there is no one with disabilities in our organisation…So you want to address that challenge of women. So, do you think that a woman with disability and a woman without disability have the same challenge, are they adequately treated? Oh, I haven't been taught about it. This is the answer of most people.”* |
| *PCP215, NGO representative, Bahir Dar* | *“And in the best case scenario is when you have these beneficiaries, these parents who feel empowered, they feel so empowered that in one of our partners, they have become volunteers to go around the town and try to identify other children for the programmes. They can benefit as well. And so that the families don't feel stigmatised and so they know that there's help available, because a lot of times in the places where we work, people find out about our projects by word of mouth. So it's quite common.”* |
| *PCP1539, caregiver, Addis Ababa* | *“I have gained a lot from my meeting with them. My daughter has a serious problem, but many parents have children that have more serious problems than her. We have become family, and I sympathise with them. We still meet once a month. We have become like sisters [the mothers]. We visit each other when a member gives birth to a child. We meet and discuss many things. I have learned a lot from my meeting with them.”* |
| *PCP1408, caregiver, Addis Ababa* | *“I want to do many things [to change my life if his health condition improves]. I want to finish the work I started doing to help my brothers. Though I have improved a lot, I used to be upset that I couldn't do anything but sit in the house. Now that he is going to school, I can bring some things from the market and sell them in retail.* |
| *PCP1219, caregiver* | *“I don't have much information, but I don't think that parents [of slowly developing children] who live abroad face the same problems as those who live in this country. I think that these people will have better lives if everyone does everything possible to support them.”* |
| *PCP216, NGO representative* | *“From an NGO, an outsider point of view, it's not always easy to work in Ethiopia…You can't really just go with things, you have to convince people…sometimes things go very slowly because you can only do it through the government system. And it's tough at times, because it means you move slow. But it also means that things go the Ethiopian way…And also, there's more protection in Ethiopia. And sometimes that doesn't help people and children. And at other times it does because you can't just get people coming in doing crazy things basically. And so I think at the end of the day, it is useful because it brings in knowledge, it brings in expertise, it brings in funds, it brings in materials, but, I think there is enough protectionism basically in Ethiopia, to not let it grow wild and become useless to the community.”* |
| PCP225, NGO representative | *“So it really helps to have me or someone [from the US] here that's helping to oversee it [the NGO]. Because there's too much temptation… I don't want to say that….in a country where there's so much poverty, there's a lot of temptation for people to use the money in the wrong way. So it's very helpful to have someone who's not going to be motivated by money or who's not in poverty or desperate. So I think just knowing who's running the place is very helpful for our donors.”* |
| *PCP212, NGO representative, Bahir Dar* | *“We can change [the life of] those person with disabilities, simply, activity we feared the dependency syndrome. If we provide some money for them in the beginning, they will share and disappear. Yeah, but still within two years, without any financial support, they are binded [bound], actually themselves have their own law [on how they support one another] and that makes them very strong to stay together. So after two years we shall provide some money...”* |
| *PCP1455, caregiver* | *“More than anything else, I want to be able to send him to school. I would cut my monthly expenses and do everything possible to send him to school if I had money.”* |
| *PCP0051, caregiver* | *“I don't do anything. I just sit [in the house] with my son. I used to wash clothes in people's houses. I met his father when we worked as daily laborers. We are not together now. I never did the same work after I faced this problem [the DD]. I didn't work for nine years. It is hard to take care of the kid and do work.”* |
| *PCP0935, caregiver* | *“I worry that people could follow her when she goes to the toilet and cause harm to her. I used to wash her when she used the toilet in the past. I don't do that now, and I tell her not to show her private parts to anyone because I fear that she could face a problem as a female... We live in a condo[minium], and there are daily labourers downstairs. Once, she told me that one of the daily labourers asked her to buy bread for him. I didn't like that. She said that he was reclining when he told her to buy bread for him. The bakery is nearby and he could have bought the bread by himself. I went downstairs and told him not to give her orders again. Because she is female, I always worry that people might harm her.”* |
| *PCP1315, caregiver* | *“He is only seven years old now. He was healthy and normal when he was born. I left the house when he was one year and nine months old because he [my ex-husband] performed domestic abuse on me. I left him [the kid] with the maid, and I went to Hawassa. I have a cousin in Hawassa. I didn't go to my parent's house because they didn't like him [my ex-husband] from the start…I returned [to my ex-husband] with my brother and my aunt after ten days. He [my son] was dormant [passive] when I met him. I thought he would run towards me because I missed him so much. However, he was inactive when I entered the house. He was very active in the past. I remember that he [my husband] beat him the day I left the house after we fought. I think I have seen him [my son] when he fell with his face to the ground as I escaped from my ex-husband and left the house. I have heard people talking about that, and I believe that his illness is directly related to what happened to him at that time.”* |
| *PCP0051, caregiver* | *“I am always upset. I always feel sad and ask God why he gave me this kind of child. I don't feel well, and I feel sick in my breast. I am always unhappy about my children's lives...I think it is the work of Satan.”* |
| *PCP115, clinician, Addis Ababa* | *“You don't know if a parent is telling you the truth or not. How can you? Everybody wants to please you. So when we do the post training, and we do pre and post training, and when you ask them, did you benefit anything from this? They tell you oh, yes, a lot. But then when you check with the school, sometimes what parents are doing is telling you that no, nothing has changed, everything is the same.”* |
| *PCP0232, caregiver* | *“At first, I thought that they would change his life immediately. Then I wanted to quit because it wasn't like I expected, but I continued taking the training because I liked the way she explained things.”* |
| *PCP111, clinician* | *“…we [clinicians] try to empathise with them [caregivers], give information, and we encourage them to support each other. So in the support group, we don't take an active role. Instead, we facilitate and we just want it to be about them and so to share experiences, the mothers meet every month and they share experiences.”* |
| *PCP221, NGO representative* | *“Parents with children with disability have to be empowered so that they can form their organisations to ask [claim] their rights, the ultimate goal is that it’s not a charity, they have to ask their right, from development agents or governments, or multilateral agencies, they have to ask their right.”* |
| *PCP221, NGO representative* | *“Some of them [mothers] don’t even have work, we try to get them work so that they can support their children, they have to have their living, and livelihood to support their children and according to our good communication there are many schools that try to give them jobs as sanitary workers or cleaners so that they can be able to feed their children.”* |
| *PCP219, NGO representative* | *“We believe that persons with disability…are rational…That means that they can be involved with any business that they want. The model that we follow is we try to make them generate income, save… and they will take loans from the association and they become involved in any kind of work but usually farming… or selling, opening shops… also mobile maintenance or petty trade, the common livelihood programme that you see.”* |
| *PCP217, NGO representative* | *“International NGOs, it’s very prominent here, and why is it needed? Because the country is very much divided based on ethnicity and religion and international agencies have a fair approach, we don’t discriminate, it’s for everyone…when it comes to national authority it’s not like that…they don’t have resources available, to address ongoing needs and crises…So three categories: ensure fair service provision, build capacity of local service providers, bringing resources to address the needs which authorities are not able to address. All international agencies are overstressed in Addis, in the countryside is different, the country doesn’t have enough capacity and resources.”* |
| *PCP114, clinician, Addis Ababa* | *“People knowing their professional boundaries, and doing something only they know and they can, you know, like, not somebody jumping and saying, I can do the speech therapy, there's somebody jumping and saying, I can do occupational therapy and so on. Not that kinds of things, somebody being trained and doing all the things...”* |
| *PCP215, NGO representative* | *“They [the NGO] know that this [intervention] has been funded by us for so many years. Our expectation would be that eventually they would like it so much, and they would feel so proud of it and they would want ownership of the programme that they would start paying for it. But economically, why would they? If they know someone else is there willing to give them funding for this, what’s the push? To start paying for it on your own, and if that funding leaves, do they feel attached enough? … And even if they believe the government should fund them, does the government believe they should fund these efforts? I think there’s a lot of unknowns when it comes to that, but I think the biggest recognition from the government is that they know they have money coming in. They know they have resources of international income. And I think sometimes, that takes of lot of weight and pressure off them to take up ownership for programmes that are being funded by international actors, regardless of where they come from.”* |
| *PCP1315, caregiver* | *“He is a small kid now, and nobody will take him seriously if he does anything wrong. However, what will happen to me in the future? How will I control him in the future? God forbid, he could beat me when he grows up and becomes strong. He could cause many problems [to me] and other people.”* |
| *PCP218, NGO representative, Addis Ababa* | *“They [families] can't even afford to feed their children once in a day. So they're forced to give them to some NGOs… but after a long time, they go and knock the door of that NGO to check whether their children are there or not…if they [caregivers] cannot afford their [children’s] food, clothes, their education, health…so sometimes they put them in the front door of the NGO [out of desperation]…”* |
